# Preparation and Characterization of Cellulose Triacetate as Support for Lecitase Ultra Immobilization

**DOI:** 10.3390/molecules22111930

**Published:** 2017-11-16

**Authors:** Francielle Batista da Silva, Wilson Galvão de Morais Júnior, Cleuzilene Vieira da Silva, Andressa Tironi Vieira, Antônio Carlos Ferreira Batista, Anízio Márcio de Faria, Rosana Maria Nascimento Assunção

**Affiliations:** 1 Laboratory of Polymers Recycling, Chemistry Institute, Federal University of Uberlândia, Uberlândia 38408-144, MG, Brazil; fran_ciellesilva@yahoo.com.br (F.B.d.S.); cleuzilene@gmail.com (C.V.d.S.); 2 Chemical Engineering Faculty, Federal University of Uberlândia, Uberlândia 38408-144, MG, Brazil; wjunioreq@gmail.com; 3 Faculty of Integrated Sciences—FACIP, Federal University of Uberlândia, Ituiutaba 38304-402, MG, Brazil; dessaqtironi@yahoo.com.br (A.T.V.); flashantonioc@gmail.com (A.C.F.B.); anizio@ufu.br (A.M.d.F.)

**Keywords:** cellulose triacetate, immobilization, Lecitase ultra, methanolysis, soybean oil

## Abstract

The use of polymers as supports for enzyme immobilization is a strategy that enables to remove the enzymes from a chemical reaction and improve their efficiency in catalytic processes. In this work, cellulose triacetate (CTA) was used for physical adsorption of phospholipase Lecitase ultra (LU). CTA is more hydrophobic than cellulose, shows good performance in the lipases immobilization being a good candidate for immobilization of phospholipases. We investigated the immobilization of LU in CTA, the stability of the immobilized enzyme (CTA-LU) and the performance of CTA-LU using soybean oil as a substrate. LU was efficiently immobilized in CTA reaching 97.1% in 60 min of contact with an enzymatic activity of 975.8 U·g^−1^. The CTA-LU system presents good thermal stability, being superior of the free enzyme and increase of the catalytic activity in the whole range of pH values. The difference observed for immobilized enzyme compared to free one occurs because of the interaction between the enzyme and the polymer, which stabilizes the enzyme. The CTA-LU system was used in the transesterification of soybean oil with methanol, with the production of fatty acid methyl esters. The results showed that CTA-LU is a promising system for enzymatic reactions.

## 1. Introduction

Cellulose acetate (CA) is an esterified cellulose derivative widely known for its numerous applications in membrane separation processes, production of matrices for controlled drug release (membranes or particles), protection of optical films, cigarette filters, among other applications [1,2,3,4]. Due to processability and biodegradable potential, an interesting field for CA applications is its use as a matrix for incorporation of bioactive species, by occlusion in the matrix or by physical adsorption [5,6,7].

**Figure 1:**
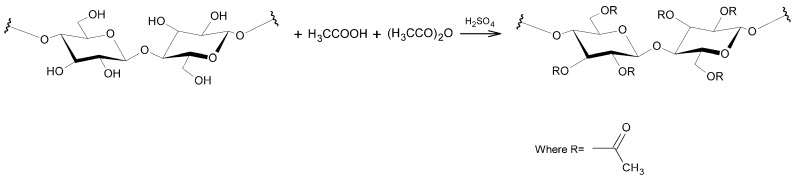
Scheme of acetylation of cellulose.

This polymer can be produced from dissolving pulp or cellulose obtained from alternative lignocellulosic sources, through the reaction of cellulose with acetic anhydride and acetic acid catalyzed by sulfuric or perchloric acid. Figure 1 shows a scheme of the cellulose acetylation reaction. CA is formed by the modification of the hydroxyl groups of each glucose unit with an acetyl group. The average number of acetyl groups per glyosidic unit, or degree of substitution (DS), can be varied from 0 in the case of cellulose to 3 in the case of cellulose triacetate (CTA) and may also be 2.0–2.5 when cellulose diacetate (CDA) is formed [8]. According to Zhou and collaborators [9], cellulose acetylation dramatically alters the surface characteristics of the product by altering the strongly hydrophilic nature of the cellulose by decreasing its polarity. As the degree of substitution is increased, there is a consistent increase in the hydrophobic character of the material obtained.

The use of natural supports, such as cellulose, for the immobilization of enzymes is advantageous since cellulose and its derivatives, particularly esterified ones, are biocompatible and present a balance of the hydrophilic/hydrophobic character important for effective enzyme adsorption and catalytic activity [10]. Considering that cellulose acetate has good processability, biocompatibility and possibility of changing the hydrophilic/hydrophobic character through acetylation/deacetylation [9], this polymer presents important properties for its use in the immobilization of bioactive species such as enzymes.

Several commercial immobilized enzymes have been used for various applications, including biodiesel production. Among the most cited are Novozyme 435 [11,12,13,14,15] and Lipozyme RM IM [16,17]. In contrast to the use of commercial biocatalysts, the development or modification of supports for the immobilization of enzymes (commercial or not) is an option that can minimize costs and even optimize reaction conditions depending on the enzymatic activity [18]. Research carried out using supports of mixtures of polylatic acid, chitosan and polyvinyl alcohol [19], cellulose nanocrystals [20], cellulose ester derivatives such as cellulose acetate (CA), cellulose acetate propionate (CAP) and cellulose acetate butyrate (CAB) films [21] were found in the literature. Moreover, Kosaka et al. [22], found that cellulose acetate films could serve as specific supports for lipases, when investigated the adhesion of bovine serum albumin (BSA) and lipase onto the cellulose esters films to evaluate the possibility of applying such films as selective support for biomolecules.

Despite the efficiency of lipases, and enzymes in general, factors such as cost and stability limit the use of enzymatic processes. Enzyme immobilization is a technique that minimizes this problem, since it allows reuse of the immobilized enzyme, an efficient separation of the reaction medium and is often able to improve enzyme activity [23]. Enzyme immobilization also can improve selectivity of proteins. Using different immobilization protocols is possible conferring different rigidity and distorting of enzyme and different physical properties [24,25,26]. Moreover, immobilization of enzymes can minimize inhibition effects, distorting or blocking active center of enzymes and reducing inhibition caused by some substrate, component of the bulk medium or product of reaction [27].

The simplest form of lipase immobilization is by adsorption onto a solid support via intermolecular forces. Physical adsorption is a popular technique because it is easy to perform, inexpensive and requires only mild chemical conditions [28]. An interesting fact about lipase is that, while its substrates are hydrophobic lipids, it is soluble in water, been fairly hydrophilic proteins with an hydrophobic area surrounding the catalytic site [29]. This means that the catalytic site is hidden within the structure of this monomeric protein which is exposed at the interface of the lipid-water interface. This feature is called interfacial activation. This mechanism suggests the presence of a lid that, when closed, covers the catalytic site and makes the enzyme inactive, but in the presence of the interface, undergoes a conformational change and opens to reveal the active site [30]. Appling a hydrophobic support as support for lipases, that resembles the surface of the drops of substrates, and low ionic strength, the enzymes become selectively immobilized on these supports. Other water soluble proteins are not adsorbed on the support under these conditions. Immobilized enzymes usually exhibit a significantly enhanced enzyme activity by the interfacial activation [31,32]. Lecitase Ultra (LU) is an artificial phospholipase developed primarily for degumming processes [33]. This enzyme is obtained from the fusion of the lipase genes of *Thermomyces lanuginosus* (for good stability) and of the phospholipase *Fusarium oxysporum* (to obtain phospholipase activity). In some respects, it behaves as a standard lipase with the ability to be adsorbed onto hydrophobic surfaces of low ionic strength (e.g., hydrophobic supports) and exhibits broad specificity [34]. This enzyme is known to have sn-1,3 regiospecificity in relation to triacylglycerol [35]. Lecitase ultra has been largely used for degumming oil [35,36,37,38,39], but also for diacylglycerols and monoacylglycerols production [33,40,41,42], enantioselective reactions [43,44], and in methyl esters production [45,46]. Many papers studied immobilization of this enzyme and improve of stability and activity of this phospholipase [34,47]. There are few studies dealing with the production of methyl esters from enzymatic transesterification using LU phospholipase [45,46].

In this study, the immobilization of LU phospholipase was performed in a CTA suspension, used as a support, via physical adsorption. Although hydrophobic characteristics that favors the physical adsorption of lipase, few studies have been carried out using CA for lipase immobilization [48,49].

The objective of this work is to prepare a stable support from Kraft pulp that can immobilize LU phospholipase by physical adsorption and to employ the immobilized enzyme in fatty acid methyl ester (FAME) production from soybean oil.

## 2. Material and Methods

### 2.1. Materials

The enzyme Lecitase Ultra (Novozymes) was donated by LNF Latino Americana Company (Bento Gonçalves, Rio Grande do Sul, Brazil). The bleached Kraft pulp, originating from eucalyptus, was donated by Suzano (Americana, São Paulo, Brazil). Refined soybean oil was purchased at a local market. Other reagents and standards used for the synthesis and characterization of cellulosic derivatives are analytical or HPLC grade.

### 2.2. Production of CA and Characterization of the Material Obtained

CA was produced as previously described with some modifications [50]. 50.0 mL of glacial acetic acid was added to 2.00 g of Kraft pulp. The mixture was stirred for 30 min at room temperature. Then a solution containing 0.16 mL of concentrated H_2_SO_4_ in 18.0 mL of glacial acetic acid was added and stirred for 25 min at room temperature. The mixture was filtered. Acetic anhydride (64.0 mL) was added to the filtrate with stirring and the filtrate was returned to the starting flask with the material. The solution was stirred for another 30 min and allowed to stand. After 5 h, distilled water was added to the reaction medium until no further precipitate was formed. The mixture was vacuum filtered by washing with distilled water to neutral pH. The material was oven dried for 90 min at 105 °C.

The samples were characterized by absorption spectroscopy using FTIR. The samples were analyzed as KBr pellets at the ratio of 1:100 (*w*/*w*) on a Shimadzu IR Prestige-21 FTIR absorption spectrometer. Thirty-two scans with a resolution of 4 cm^−1^, in the range of 500–4000 cm^−1^ were made.

The degree of substitution of the material obtained was calculated from the ratio between the absorbances of the drawstring C=O (1750 cm^−1^) and OH (3400 cm^−1^). According to Hurtubise [51] the ratio between these absorbances is related to the percentage of acetyl groups (*AG*) of CA (%*AG*). The value of the percentage of acetyl groups relates to DS according to Equations (1) and (2) [52].

(1) $$\%AG=43.69{\left(1-e^{-0.974x}\right)}^{2.153}$$

where *x* = ratio between the absorbances of the drawstring C=O (1750 cm^−1^) and OH (3400 cm^−1^).

(2) $$DS=\left(\frac{162\times \%GA}{43\times 100-42\times \%GA}\right)100$$

Images of Kraft pulp and CA were obtained by SEM. The equipment used was a Carl Zeiss model EVO 10 MA scanning electron microscope. The samples were previously metallized with a thin gold layer using a Sputtering Leica.

### 2.3. Immobilization of LU on CTA

One gram of CTA was added to 10 mL of LU solution 30% (*v*/*v*) (335 U/mL, 5.6 mg/mL protein) in a 25 mM sodium phosphate buffer pH 7. The suspension was maintained under static conditions at 4 °C [53]. The enzymatic activities of the suspension and also of the supernatant (without the presence of the support) were measured periodically to follow the immobilization process. After immobilization, the support was washed with deionized water, vacuum filtered and stored at 4 °C for later use. The biocatalyst obtained was called CTA-LU.

Yield of immobilization (YI) was defined as the ratio between the amount of immobilized enzyme and the amount of enzyme offered to immobilization (% immobilization).

### 2.4. Enzymatic Activity Assay and Protein Dosage

The assays were performed by measuring absorbance at 410 nm, which is produced by the release of *p*-nitrophenyl palmitate (*p*-NPP). The samples were incubated in a 50 mM sodium acetate buffer at pH 5, containing 0.05% (*w*/*v*) arabic gum, 0.25% (*v*/*v*) Triton X-100 and *p*-NPP solution, consisting of 3 mg *p*-NPP/mL of 2-propanol. Aliquots of the samples (0.1 mL of free enzyme or 0.1 g of immobilized enzyme) were added to the reaction solution in a ratio of 1:9 (*v*/*v*) and the reaction was controlled for 10 min at 50 °C. After this time, samples were taken at 92 °C for 2 min and then in the ice bath with the addition of saturated sodium borate solution. The absorbance was measured (PG Instruments T70 Spectrophotometer, Lutterworth, UK). One unit of enzyme is equivalent to the release of 1 μmol of *p*-nitro phenol (*p*-NP) (ε = 7.93 umol·cm^−1^) per minute by 1 mL of free enzyme or per gram of immobilized enzyme (weight of matrix included). The methodology is an adaptation from Pencreach and Baratti [54]. 

The protein dosage of the enzyme was determined according to the method of Bradford [55] using a previously made calibration curve.

### 2.5. Biochemical Characterization of Lecitase Ultra Immobilized in CTA (CTA-LU)

#### 2.5.1. Effect of pH and Temperature on Stability of Soluble LU and CTA-LU

Aiming to evaluate pH effect on activity of LU, free and immobilized lipase were kept at room temperature for 2 h in a 50 mM sodium citrate buffer (at pH 3, 4 and 5); a 50 mM phosphate buffer (at pH 6, 7 and 8); and a 50 mM sodium bicarbonate buffer (at pH 9 and 10). Enzyme activity was measured according to the methodology described in Section 2.4. 

Free and immobilized LU were diluted in 50 mM phosphate buffer at pH 7, then incubated at different temperatures (30, 35, 40, 45 and 50 °C) for 8 h, for evaluate thermal stability of lipase. The enzyme activity was measured according to Section 2.4. 

The thermal inactivation of the enzyme was evaluated through a pseudo first order kinetic model according to Equation (3) [56]:

(3) $$ln\left(\frac{A_{t}}{A_{0}}\right)=-k_{d}\times t$$

where *A_t_* is the enzyme activity at treatment *t*, time (h), *A*_0_ is the initial enzyme activity and *k_d_* is inactivation rate constant at the temperature studied. 

The *k_d_*, for different temperatures were estimated by linear regression analysis of the natural logarithm of residual activity versus time. 

#### 2.5.2. Effect of Organic Solvents on Stability of CTA-LU

The effect of some solvents (methanol, ethanol, *iso*-propanol, *n*-hexane) was studied on activity of immobilized lipase. The immobilized lipase was separately incubated with each of the selected solvents (added in 50 mM phosphate buffer pH 7) at room temperature for 30 h. The enzyme activity was evaluated according to Section 2.4.

#### 2.5.3. Desorption of LU from CTA

CTA-LU samples were added to solutions containing Triton X-100 in a 25 mM sodium phosphate buffer at pH 7.0 and 25 °C, at concentrations of 0.02%, 0.2%, 0.5% and 1.0% (*v*/*v*). The samples were mechanically stirred for 50 min and the enzymatic activities were calculated as described in Section 2.4.

### 2.6. Reusability of CTA-LU

Derivative reuse was performed in batch assays by incubating 0.1 g of CTA-LU with 0.90 mL of *p*-NPP solution (prepared as previously described). After 10 min of reaction, samples were taken at 92 °C for 2 min and then in the ice bath with the addition of saturated sodium borate solution. The released *p*-NP was measured at 410 nm. After each cycle, CTA-LU was washed with 25 mM sodium phosphate buffer pH 7.0, filtrated and added to a new hydrolysis cycle with new substrate solution. The activity after each cycle was expressed in relation to the activity after the first cycle.

### 2.7. Methanolysis Product of Soybean Oil Catalyzed by LU Immobilized in CTA

Transesterification reactions were performed with soybean oil, at a molar ratio of 1:4 (oil/methanol), 20% (% by oil wt.) of CTA-LU, 15% (% by oil wt.) of water and 3.3% (by oil wt.) of *n*-hexane. Reactions were carried out under magnetic stirring (250 rpm), at 35 °C and finalized after 24 h. The samples were centrifuged and the upper phase was collected and analyzed by chromatography. No purification step was performed after the reaction.

The molar ratio of soybean oil was calculated by Equation (4):

(4) $$M_{oil}=3\left(\frac{\sum \%FA\;\times M_{FA}}{\sum \%FA}\right)+38.04$$

where *M_oil_*: molar mass of soybean oil; %*FA*: molar percent of fatty acids; *M_FA_*: molar mass of fatty acids; 38.04: difference between the molar mass of glycerin and the three water molecules that replace glycerin.

Analyses of fatty acid methyl esters (FAMEs) were conducted by gas chromatography in an Shimadzu gas chromatograph (GC2010-Plus) equipped with a flame ionization detector (FID) and RTX-WAX capillary column (30 m × 320 μm × 0.25 μm). The split ratio was 1:10 and helium was used as the carrier gas. The amount of sample injected was 1 μL and the total time of analysis was 15 min. An analytical standard FAME mix (C4–C24) from Supelco was used to identify the peaks at different retention times. Peak identities were confirmed by analyzing the synthesis product in a gas chromatography system equipped with a Shimadzu mass spectrometer, GCMS-QP2010 (Columbia, MD, USA) model. The FAME content was calculated using the compensated normalization method with internal standardization based on the European standard EN 14103. Methyl heptadecanoate was used as the internal standard in this analysis.

## 3. Results and Discussion

### 3.1. Production and Characterization of CA

For production of cellulose acetate, the homogeneous process was used. In the homogeneous process, in which non-swelling agents are not employed, cellulose triacetate (CTA) is solubilized in the reaction medium as it is produced. An important characteristic of cellulosic derivatives is the possibility of mediating, by chemical modification, their hydrophilic/hydrophobic character. This property is important because lipases have high affinity for hydrophobic supports and in various cases immobilization in this type of support increase activity of enzymes [29]. In addition, cellulose acetate can be obtained from any cellulosic source if the residues are purified and withdrawn from lignin and hemicelluloses [1,8,50,57,58,59].

**Figure 2:**
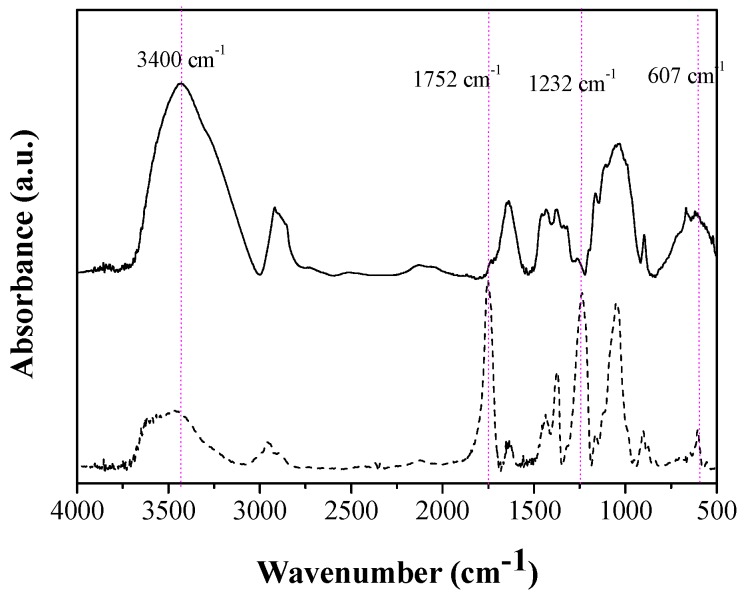
Infrared spectrum obtained for Kraft pulp (straight line) and cellulose triacetate (dashed line).

The CA synthesis was confirmed by FTIR analysis. Figure 2 shows infrared spectra of the Kraft pulp and CA obtained.

It can be observed from Figure 2 that cellulose was acetylated. This is confirmed in the CA spectra by the decrease in the peak intensity close to 3400 cm^−1^, the associated stretching of OH groups and the appearance of the ester carbonyl peak at 1752 cm^−1^ due to O=CO-CH_3_ stretching of acetate and the CO stretching of acetyl groups at 1232 and 607 cm^−1^. This is the strongest evidence that the material has been acetylated, since the other peaks are common to both materials. The degree of substitution obtained from the ratio between the absorbance (Equations (1) and (2), Section 2.2) of the C=O stretching peaks and the OH stretching bands was 2.7, indicating that the material obtained is cellulose triacetate (CTA). Moreover material obtained presents solubility in dichloromethane [60,61,62].

**Figure 3:**
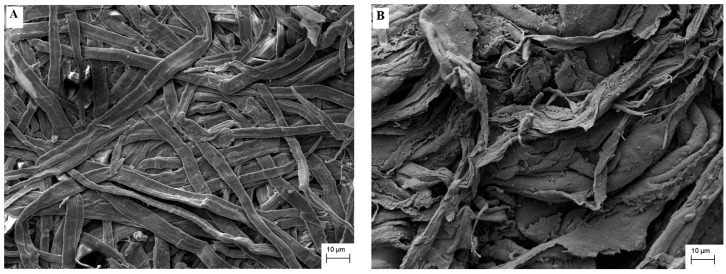
Scanning electron microscopy for samples of (**A**) Kraft pulp and (**B**) CTA.

The images obtained by scanning electron microscopy (SEM), presented in Figure 3, indicate the structure modification when comparing Kraft pulp (Figure 3A) with CTA (Figure 3B). There is a modification in the structure of the fibers of the feedstock when the material is acetylated. During synthesis, fiber swelling occurs due to the presence of glacial acetic acid and hydrolysis, which may occur due to the presence of the sulfuric acid catalyst. These processes modify the morphology of the fibers, which become more disorganized and undergo a defibrillation process.

The chemical modification of the Kraft pulp through the reaction of the hydroxyl groups of the cellulose with the acetic groups of the acetic anhydride leads to the production of the cellulose triacetate, a cellulosic derivative more hydrophobic than the original cellulose. Thus, the CTA would be suitable for the immobilization of lipases by physical adsorption, since it resembles the substrates of the lipases, allowing them to readily attach to the support.

### 3.2. Enzyme Immobilization

**Figure 4:**
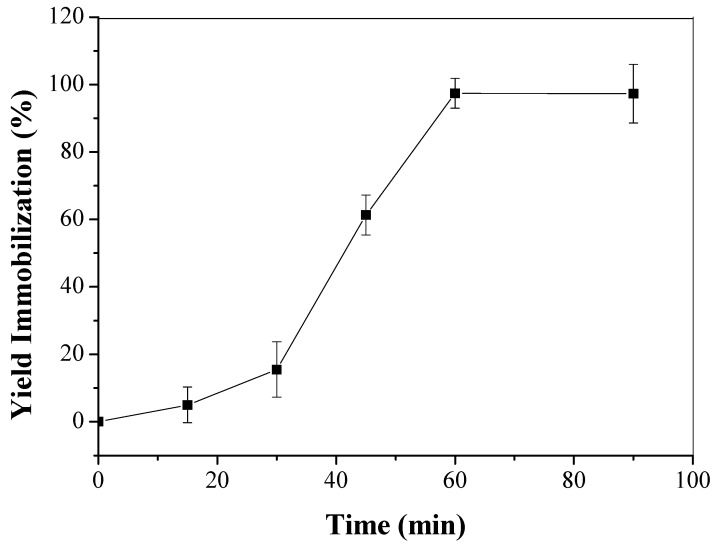
Yield of immobilization versus incubation (immobilization) time for LU in CTA support. The error bars are derived from triplicates.

LU was immobilized on CTA with a maximum immobilization yield of 97.1 ± 4.4% after 60 min (Figure 4) presenting a specific activity of 975.8 U·g^−1^ support. The immobilization of LU in CTA probably occurred via interfacial activation. This is a peculiar mechanism of lipases, which relates to the open form of the lipase when in contact with a hydrophobic surface in low ionic strength medium [63].

**Figure 5:**
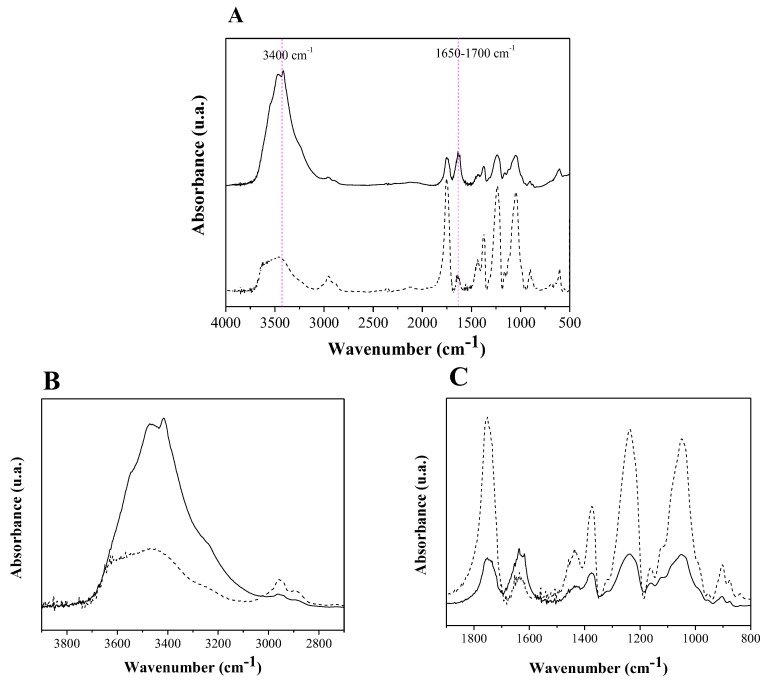
Spectrum in the infrared region for CTA (dashed line) and CTA-LU (straight line) (**A**) complete spectrum; (**B**,**C**) cutting of the most important regions of the spectrum.

Figure 5A shows the spectra in the infrared region for the CTA and CTA-LU. The biocatalyst retains much of the characteristics of the original CTA, except for the O-H stretch region and a slight change in the region between 1660 and 1500 cm^−1^. The band at approximately 3400 cm^−1^ exhibits a typical O-H stretching profile attributed to adsorbed water and N-H stretching, typical of the polypeptide structure of the enzymes, best seen in Figure 5B. The polypeptides have a characteristic structure related to the presence of the amide group, which can be classified as amide I and amide II. The amide-related band I is attributed to the carbonyl stretch, C=O, of the (CONH) group and appears between 1700 and 1650 cm^−1^, as can be seen in Figure 5C. The band at 1650 cm^−1^ can also be influenced by the presence of water, since the angular deformation of a water molecule appears between 1640 and 1645 cm^−1^ [64].

Although the contribution of the bands of the enzyme is small, it is sufficient to indicate their immobilization in CTA. This technique is usually used to confirm enzyme presence in synthetized supports [65,66].

**Figure 6:**
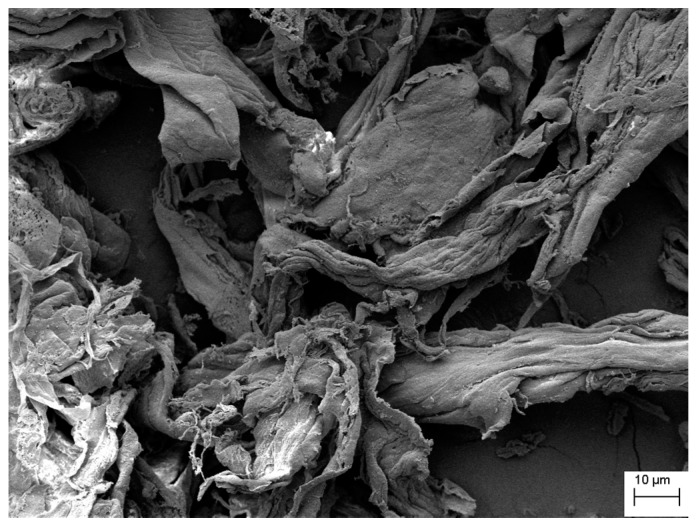
SEM image of CTA-LU.

Figure 6 shows an SEM image of CTA-LU sample, CTA with immobilized enzyme. The sharp structure of the fibers is apparent in the original cellulose (Figure 3A) and appears in a disorganized and more compacted form in CTA (Figure 3B). The structure is less evident and more than immobilized enzyme). This behavior may be associated with the processing of immobilization where possible swelling and drying processes lead to aggregation of the CTA.

### 3.3. Biochemical Characterization of CTA-LU

#### 3.3.1. Temperature and pH Effects on Stability of Soluble LU and CTA-LU

**Figure 7:**
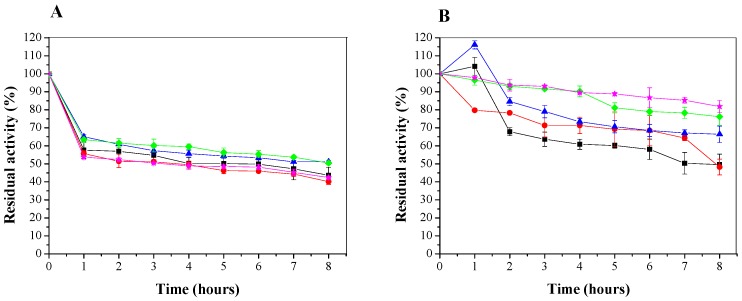
Thermal stability of free (**A**) and immobilized lipase (**B**) at temperatures of 30 °C (▲), 35 °C (♦), 40 °C (⋆), 45 °C (∎) and 50 °C (●) in 25 mM sodium phosphate′ buffer, pH 7. The error bars are derived from triplicates.

The thermal stability of Lecitase Ultra and immobilized lipase were studied by measuring residual enzyme activity as function of temperature for 8 h. Figure 7 shows the curve patterns of residual activity versus time for different temperatures (30, 35, 40, 45 and 50 °C).

Figure 7A shows the effect of temperature on the activity of free lipase. It was observed that with one hour of incubation at 30 and 35 °C, the enzyme undergoes an inactivation of approximately 35%, with a loss of activity close to 50% after 8 h. At 40, 45 and 50 °C, the inactivation is shown to be 45%, 43% and 47%, respectively.

The effect of temperature on the activity of immobilized lipase was also studied (Figure 7B). At 30 °C, it was noted that in the first hour of the experiment, the immobilized LU in the CTA support underwent activity increase and enzyme presented a final residual enzyme activity of 66% after 8 h of incubation. At 45 °C, similar behavior was observed: an activity increase in the first hour of the experiment, showing a final residual enzymatic activity of 48%, which is close to that obtained at 50 °C. Looking at the results, it is noticeable that the immobilized enzyme is more stable at 35 and 40 °C with an inactivation of 24% and 19.3%, respectively, after 8 h.

The increase in temperature generally causes an increase in reaction rate, with or without catalysts. However, enzymes are biological systems of complex nature, with catalytic activity dependent on their tertiary structure and this must be taken into account.

Table 1 shows the values of *k_d_* for free and immobilized enzyme. In both cases, changes in *k_d_* values are observed for the evaluated temperatures. The changes in *k_d_* values are related to the occurrence of two competitive processes: (i) the increase of the catalyzed reaction rate due to the increase in temperature and (ii) at high temperatures, the reduction of the reaction rate due to inactivation of the enzyme. Thus, the increase of *k_d_* indicates that the inactivation of the enzyme is predominant at the evaluated temperature and in this condition the enzymatic reaction will occur slowly.

For free enzyme, in Figure 7A and in Table 1, the values of residual activity (%) are close, as well as the data of *k_d_* undergoes minor changes in the incubation temperatures between 30 and 35 °C. This result indicates that, for this condition, the stability of the enzyme is maintained. For temperatures higher than 40 °C, there is a decrease in residual activity (%) accompanied by a small increase in *k_d_* values, which, although obtained from a fragile linear adjustment of the experimental data, shows a tendency to deactivate the free enzyme. This effect can be confirmed when we observe the data obtained for immobilized enzyme. The effect of the presence of the support is evident, since the linear fits for the calculation of the deactivation constant present less dispersion and a better tendency to linearity. In Figure 7B and in Table 1, an increase in the residual activity that reaches the best condition at 40 °C is observed at temperatures above this the system presents a more pronounced drop in the residual activity (%) and an increase of *k_d_* values, indicating that the system becomes thermally unstable. Since the CTA-LU system presents better adjustment of the experimental data for incubation temperatures in which the system is more stable and loses this condition with the increase of the incubation temperature to values higher than 40 °C. In this sense, the immobilization of the enzyme in the support stabilizes the active site and avoids the change of the tertiary structure in the considered temperature range.

The results obtained in this work were similar to those of Mishra et al. (2009), studied the thermal stability of purified LU by measuring residual activity after incubation at a given temperature for 1 h. In the study, the incubation temperature was varied between 30 and 70 °C. The results showed that the enzyme is stable up to 50 °C and loses its activity rapidly at 60 °C, with a residual activity of about 10%. For the incubation temperatures between 30 and 40 °C, the residual activities are close and presents values between 80% and 90%.

**Figure 8:**
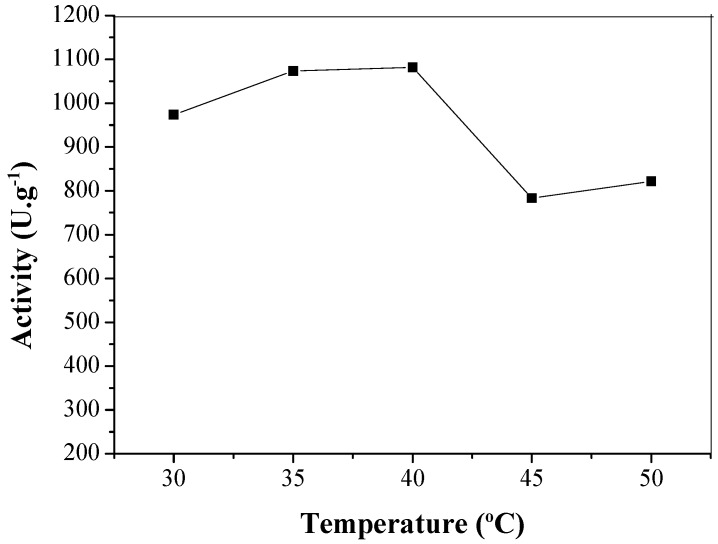
Temperature optimum of CTA-LU with lipase activity measured at pH 7.

The enzyme activity was dependent on temperature, as illustrated in Figure 8. LU immobilized in CTA exhibited maximum activity at 40 °C, the same temperature described in literature for free enzyme [35].

**Figure 9:**
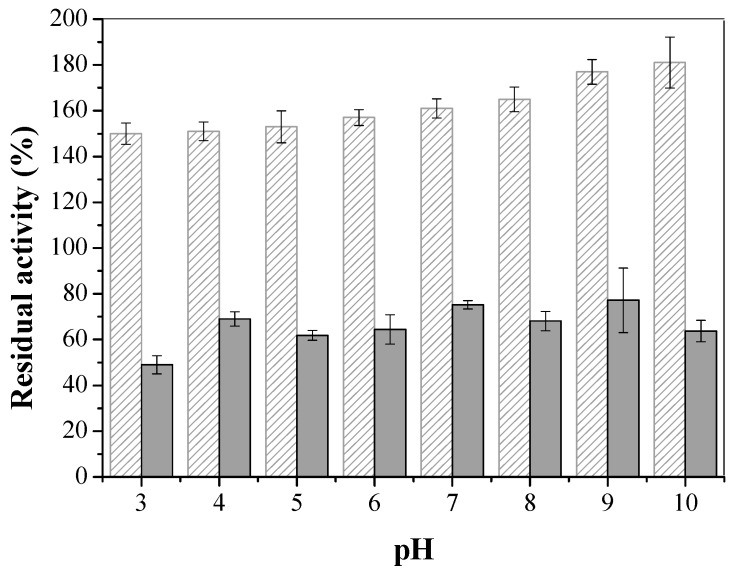
Residual activity (%) related to pH variation for free LU (solid) and immobilized LU (striped) in CTA. The error bars are derived from triplicates.

The results of the pH study, shown in Figure 9, indicate that compared to soluble LU, the immobilized lipase underwent activity increase at all studied pHs. Moreover, the highest pH values (more alkaline) this effect is more accentuated. LU immobilized on Octyl-Sepharose also showed increased activity with an increase in pH [67].

#### 3.3.2. Effect of Organic Solvents on Stability of CTA-LU

**Figure 10:**
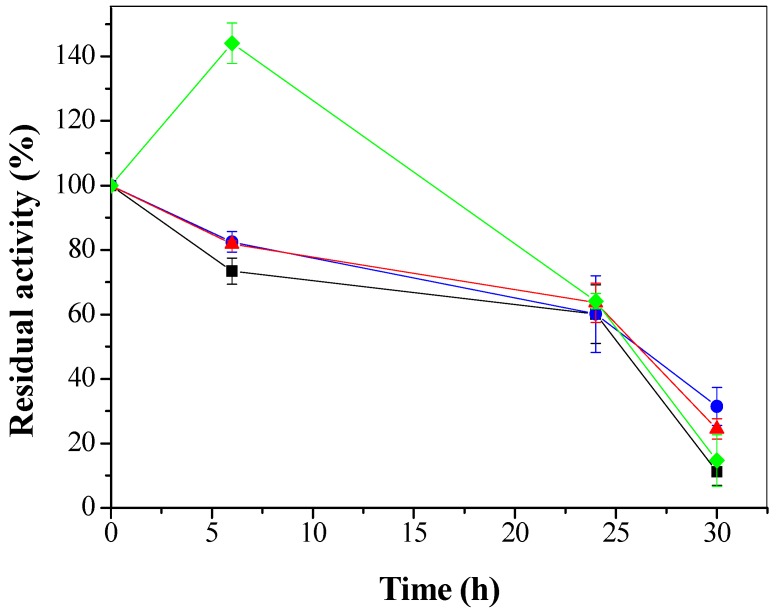
Residual activity (%) in relation to the presence of solvents: methanol (∎), ethanol (●), propanol (▲) and hexane (♦) in the immobilized LU for CTA. The error bars are derived from triplicates.

The effect of organic solvents on the stability of CTA-LU using ethanol, methanol, propanol and hexane at a final concentration of 50% (*v*/*v*) was studied under mechanical stirring for 30 h at 25 °C. Figure 10 shows the stability profiles of the CTA-LU in the presence of the different organic solvents. CTA-LU underwent activity increase in the presence of hexane after 6 h incubation. At 24 h, the immobilized LU presented similar relative activity in all the studied organic solvents, with an inactivation of approximately 38%.

The use of organic solvents in the enzymatic synthesis of FAME reduces system viscosity and aids mass transfer, improving the mutual solubility of hydrophobic triglycerides and hydrophilic alcohols, protecting the enzymes from denaturation by high concentrations of alcohols and ensuring transesterification with the addition of alcohol in only one step. Some organic solvents used for this application are isooctane, *n*-heptane, petroleum ether, *n*-hexane, cyclohexane and *tert*-butanol. Through the use of these solvents, an increase in the rate of transesterification is also observed [68].

#### 3.3.3. Enzymatic Desorption

**Figure 11:**
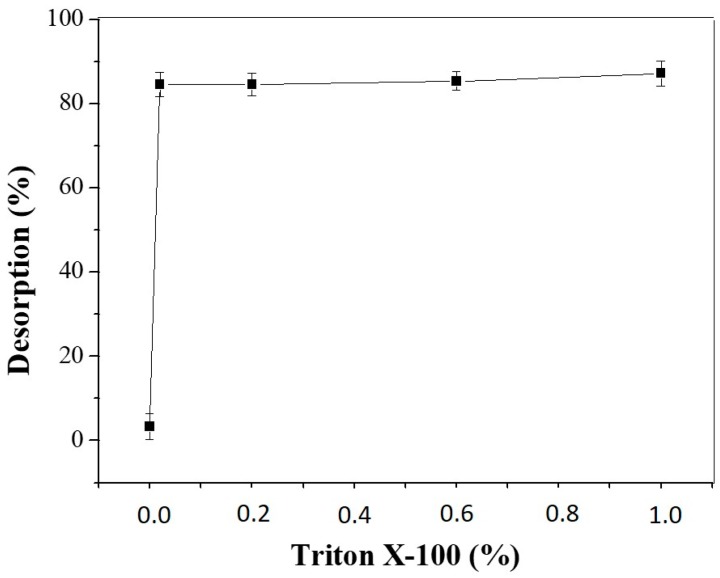
Desorption of the enzyme LU immobilized in CTA using Triton X-100 at concentrations of 0.02%, 0.2%, 0.5% and 1.0% (*v*/*v*). The error bars are derived from triplicates.

Desorption of LU was assisted by the presence of aqueous solutions of Triton X-100 at concentrations of 0.02%, 0.2%, 0.5% and 1.0% (*v*/*v*), the results can be seen in Figure 11.

It was observed that 84.5% of the lipase was desorbed from the support with a 0.02% concentration solution of Triton X-100. Increasing the concentration of the surfactant solution to 0.2%, 0.5% and 1.0%, gave only a small increase in desorption. This result shows that enzyme removal readily occurs using low concentrations of surfactant. 

Cabrera et al. [69] observed that the amount of surfactant for complete enzyme desorption depends on the type of biocatalyst produced. Depending on the biocatalyst, different concentrations of Triton X-100, ranging from 1 to 4%, were employed for complete desorption of lipase. This aspect allows to evaluate the association enzyme—support from the point of view of the intermolecular interactions and the morphology of the support [31]. Considering the intermolecular interactions, the immobilization of LU on CTA occurs in a similar way to that observed for other hydrophobic polymers.

The easy removal of 85% of the enzyme allows the support to be reused in another enzymatic immobilization. The adsorption of enzymes by supports are strong enough to use the enzyme preparation as an immobilized one, although the enzyme may be desorbed by using detergents. This fact allows the reversibility of immobilization, which is one advantage of physical adsorption [44].

### 3.4. CTA-LU Reuse

**Figure 12:**
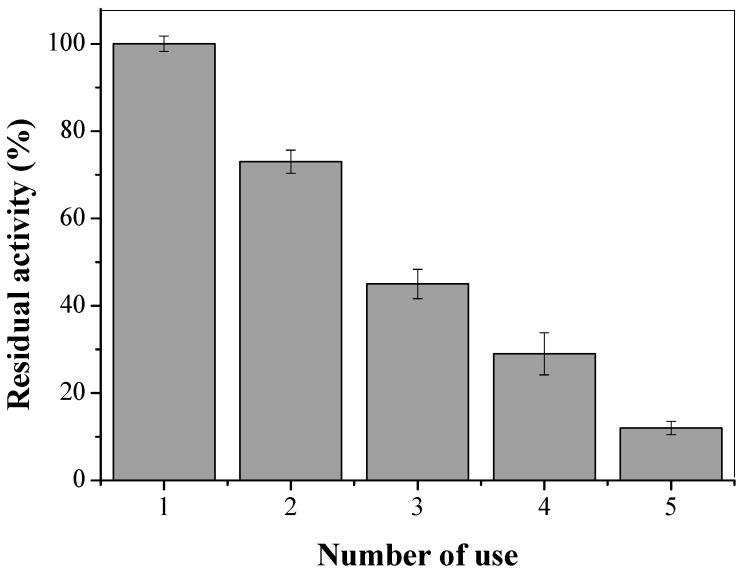
Reuse of CTA-LU on *p*-NPP hydrolysis. Reactions carried out as described in Section 2.6. The error bars are derived from triplicates.

In order to evaluate the reusability, CTA-LU was submitted to successive cycles of *p*-nitrophenyl palmitate (*p*-NPP) hydrolysis (Figure 12).

CTA-LU showed constant activity decrease after each reaction cycle, retaining more than 50% of activity up to only two cycles. The activity loss may be related to enzyme release from CTA, possible caused by detergent (0.25% *v*/*v* Triton X-100) used to prepare *p*-NPP substrate [70] and various steps of experiment. It was observed in Figure 11 that the desorption of CTA-LU is favored by the presented of Triton X-100. Turati et al. [71] also observed similar behave on immobilization of Lipase from *Penicillium* sp. Section Gracilenta (CBMAI 1583) by physical adsorption using Triton X-100 during substrate preparation. 

Immobilization of lipases by physical adsorption has a main drawback: desorption of enzymes when exposed at high temperatures, or in presence of detergents, organic solvents, or some products of reactions [70]. Some heterofunctional supports have been proposed for solve or minimize this effects, having some groups (cationic/anionic groups [72], glutaraldehyde [73], glyoxyl [74], epoxide [75]). Fernandez-Lopez [76] recently proposed the production of the physical coating of lipases immobilized on octyl-agarose (OC) by using ionic polymers to physically crosslink different immobilized lipase molecules. The strategy permitted to avoid enzyme desorption, improving enzyme stability, but maintaining the reversibility of the immobilization.

### 3.5. Methanolysis Product of Soybean Oil Catalyzed by LU Immobilized in CTA

An evaluation of the production of FAME from soybean oil using CTA-LU (195 U/g of soybean oil) as catalyst was carried out. The products of the reaction were analyzed by gas chromatography with a flame ionization detector and the identity of each peak of methyl ester was confirmed by gas chromatography coupled with mass spectrometry.

The yields of methyl ester obtained was 43.8 ± 0.6%. The FAME content was calculated using the compensated normalization method with internal standardization based on the European standard EN 14103. Methyl heptadecanoate was used as the internal standard in this analysis.

**Figure 13:**
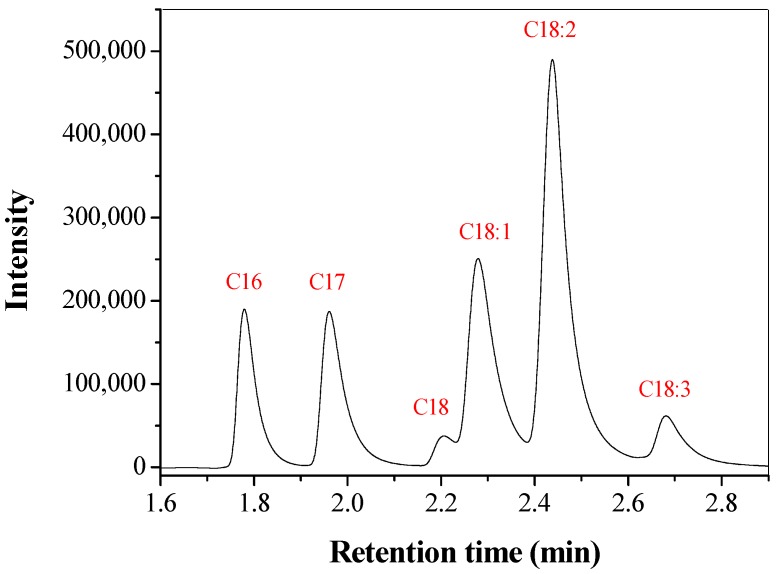
Chromatographic profile of esters formed in reactions catalyzed by LU immobilized on CTA during 24 h. Chromatographic conditions: column oven temperature: 210 °C. Split injector: 250 °C, split ratio 1:10; injected volume: 1.0 μL.

Figure 13 shows the chromatographic profile of biodiesel produced from soybean oil methanolysis catalyzed by LU immobilized in CTA resulted in 5 peaks in the chromatogram. 

It is possible to observe peaks corresponding to the main fatty acid esters of soybean oil: C16, C18, C18:1, C18:2 and C18:3 (palmitic, stearic, oleic, linoleic and linolenic acids, respectively). The chromatographic profile of biodiesel obtained from soybean oil catalyzed by LU immobilized on CTA is similar to that obtained from soybean biodiesel via alkaline catalysis [77]. This result indicates transesterification of soybean oil by CTA-LU. 

LU is an enzyme that has phospholipid and lipid activity being able to hydrolyze esters of carboxylic acids as well as phosphates of esters of fatty acids. In the literature, it is suggested that Lecitase Ultra has a single active site which possesses both lipase and phospholipase activities [44]. The enzymatic production of biodiesel depends on enzyme and media, in the case of LU, the existence of a single active site for both activities would possibly make the system even more sensitive to the medium conditions. It is important to emphasize that LU presents, under specific conditions, good catalytic capacity to produce methyl esters, similar to those observed for commercial enzymes prepared only for this purpose [46].

Although Lecitase ultra is a phospholipase, it has lipolytic activity and shown to be capable of producing methyl esters from commercial soybean oil. The % FAME obtained in this work is relatively low that any others obtained with other lipases [78,79,80]. Nevertheless, compared to results using LU in other supports, or even immobilized in another way, such production is acceptable. Tacias-pascacio and collaborators [46] recently evaluated immobilized LU in the production of biodiesel from used cooking oil. After 24 h at 30 °C with stirring, using 2 g of oil, 10% of the biocatalyst (*w*/*w* of oil), 0.25% of water (*w*/*w* of oil), 3:1 methanol:oil molar ratio and 3 mL of hexane, the maximum yield obtained was 11%, when LU was immobilized in macroporous styrene and octadecyl methacrylate, being this value lower than the 44% obtained in this work with the same solvent using CTA as support. These results suggest that the immobilized LU in CTA shows good performance in biodiesel production under the conditions tested. Anyway, studies using other sources and/or other protocols of immobilization, especially with heterofunctional groups, would be interesting in the future.

## 4. Conclusions

Immobilization of phospholipase, Lecitase Ultra (LU) in Cellulose Triacetate (CTA) opens perspectives for the application of CTA as a support for this enzyme and permits the use of this biocatalyst in several esterification, hydrolysis and transesterification processes. The immobilization of LU in CTA was efficient, reaching 97% with 60 min of contact. The adsorption process was efficient and reversible, so the enzyme could be removed from support using Triton X-100. The immobilized enzyme exhibits higher thermal stability than free lipase. This aspect was observed by shifting of the optimum temperature of the enzyme from 35 (free enzyme) to 40 °C (immobilized enzyme). The difference observed for immobilized enzyme compared to free one, in relation to temperature and incubation time, showed the efficiency of the immobilization and the stabilization of LU adsorbed on CTA. In this sense, the enzymatic activity for LU was tested using soybean oil as a substrate. The transesterification reaction with methanol occurred with production of 43.8% of FAME. Although Lecitase ultra is a phospholipase, it has lipolytic activity and shown to be capable of producing methyl esters from commercial soybean oil.

## Figures and Tables

**Table 1:** Thermal denaturation kinetic parameters for free and immobilized lipase.

	Free Enzyme		Immobilized Enzyme	
T (°C)	*k_d_* (h^−1^)	R^2^	*k_d_* (h^−1^)	R^2^
30	0.062 ± 0.0013	0.6591	0.050 ± 0.0058	0.9199
35	0.058 ± 0.0039	0.6791	0.036 ± 0.0055	0.9568
40	0.069 ± 0.0028	0.5706	0.024 ± 0.0013	0.9825
45	0.071 ± 0.0091	0.6526	0.076 ± 0.0067	0.8887
50	0.077 ± 0.0057	0.6506	0.064 ± 0.0074	0.8225
